# Factors associated with mode of colorectal cancer detection and time to diagnosis: a population level study

**DOI:** 10.1186/s12913-016-1944-y

**Published:** 2017-01-05

**Authors:** Khokan C. Sikdar, James Dickinson, Marcy Winget

**Affiliations:** 1 Community Health Sciences and O’Brien Institute for Public Health, Cumming School of Medicine, University of Calgary, G 214 HSC, 3330 Hospital Drive NW, Calgary, AB T2N 4N1 Canada; 2 Departments of Family Medicine and Community Health Sciences, Faculty of Medicine, University of Calgary, Calgary, Canada; 3 Department of Medicine, Stanford University, Stanford, CA 94305 USA

**Keywords:** Colorectal cancer, Diagnostic delay, Wait time, Primary health care, Administrative data

## Abstract

**Background:**

Although it is well-known that early detection of colorectal cancer (CRC) is important for optimal patient survival, the relationship of patient and health system factors with delayed diagnosis are unclear. The purpose of this study was to identify the demographic, clinical and healthcare factors related to mode of CRC detection and length of the diagnostic interval.

**Methods:**

All residents of Alberta, Canada diagnosed with first-ever incident CRC in years 2004–2010 were identified from the Alberta Cancer Registry. Population-based administrative health datasets, including hospital discharge abstract, ambulatory care classification system and physician billing data, were used to identify healthcare services related to CRC diagnosis. The time to diagnosis was defined as the time from the first CRC-related healthcare visit to the date of CRC diagnosis. Mode of CRC detection was classified into three groups: urgent, screen-detected and symptomatic. Quantile regression was performed to assess factors associated with time to diagnosis.

**Results:**

9626 patients were included in the study; 25% of patients presented as urgent, 32% were screen-detected and 43% were symptomatic. The median time to diagnosis for urgent, screen-detected and symptomatic patients were 6 days (interquartile range (IQR) 2–14 days), 74 days (IQR 36–183 days), 84 days (IQR 39–223 days), respectively. Time to diagnosis was greater than 6 months for 27% of non-urgent patients. Healthcare factors had the largest impact on time to diagnosis: 3 or more visits to a GP increased the median by 140 days whereas 2 or more visits to a GI-specialist increased it by 108 days compared to 0–1 visits to a GP or GI-specialist, respectively.

**Conclusion:**

A large proportion of CRC patients required urgent work-up or had to wait more than 6 months for diagnosis. Actions are needed to reduce the frequency of urgent presentation as well as improve the timeliness of diagnosis. Findings suggest a need to improve coordination of care across multiple providers.

## Background

Colorectal cancer (CRC) is the second most common cancer in men and the third most common cancer in women in Canada. Every year almost 24,000 Canadians are diagnosed with and 9,200 die from CRC [1]. The diagnosis of CRC is often not straightforward. Symptoms are often non-specific and commonly occur in benign conditions [2]. Presentation and diagnosis are therefore often delayed until the cancer is advanced [3]. Screening asymptomatic individuals can effectively detect CRC earlier; however, many nations, including Canada, have either a relatively new or no colorectal screening program [4, 5]. Early diagnosis of CRC is important because 5-year survival is much higher (90%) than that from metastatic disease (11%) [6].

A long time from symptom onset to diagnosis or treatment has been shown to contribute to cancer progression, higher stage disease and worse survival for cancer patients [7–10]. Individuals diagnosed promptly may benefit from timely treatment, which would improve prognosis and decrease patient anxiety [11].

Although there is consensus that patients should be diagnosed in a timely manner, there is little information regarding a reasonable length of diagnostic interval, that is, time from patient first presentation to the healthcare system to definitive diagnosis, or understanding of what factors affect the length of the diagnostic interval. Whether patients are diagnosed via screening or symptomatic presentation, a typical pre-diagnostic work-up may include visits to a family physician/general practitioner (GP), diagnostic imaging tests, follow-up visits and endoscopy, requiring a healthcare contacts with a variety of care providers.

The patients who present with advanced stage cancer are likely to be sicker, receive urgent care and be diagnosed more quickly than those who initially present with early stage cancer [10]. Some in this latter group may experience a prolonged diagnostic process because their symptoms are not severe or specific, or they have a chronic gastrointestinal (GI) condition that complicates diagnosing the cancer. Others that are asymptomatic may be diagnosed through screening. Screening may be conducted through a formal program or via opportunistic screening, as part of a visit for other reasons, notably for preventive care. Furthermore, the diagnostic work-up for CRC differs from most other common cancers because most CRCs are diagnosed by lower gastrointestinal endoscopy [12]. Given the complexity of the relationship between the patient’s clinical condition at first presentation, health system factors and the diagnostic interval, investigation is warranted to better understand the relationship between these factors.

The purpose of the present study was to: 1) estimate the proportion of CRC patients that are detected via an opportunistic screening visit, urgent and non-urgent symptom visits; 2) identify patient demographic, clinical and healthcare utilization factors related to mode of CRC detection; and 3) identify patient demographic, clinical and healthcare utilization factors related to time to diagnosis for non-urgent cases.

## Methods

### Study population

All residents of Alberta, Canada diagnosed in years 2004 to 2010 with malignant, invasive CRC (*International Classification of Disease for Oncology* (ICD-O) 3^rd^ edition codes C18-C20) were identified from the Alberta Cancer Registry. Individuals with a first-ever primary colorectal cancer were included. Patients were excluded for the following reasons: 1) stage 0 cancer; 2) histology that is not of colon origin (e.g., sarcomas, melanomas, lymphomas); 3) unknown stage; 4) disease was not pathologically or microscopically confirmed; 5) prior history of CRC or stomach cancer; 6) diagnosis of another cancer within 6 months prior to diagnosis of CRC; and 7) missing data that prevented identification of first visit prompting diagnostic work-up.

### Data sources and relevant healthcare encounters

Additional information obtained from the cancer registry included age and residence at diagnosis, anatomic site, and stage and date of diagnosis of tumor. The quality and completeness of the Alberta Cancer Registry has consistently been shown to be very high [13].

Health service utilization prior to and related to CRC diagnosis was obtained from three provincial administrative health databases: 1) the Discharge Abstract Database that contains inpatient data from all Alberta hospitals; 2) the Ambulatory Care Classification System Database that contains outpatient data from all Alberta hospitals; and 3) the Physician Billing Database that contains billing claims submitted by all physicians in Alberta remunerated on a fee-for-service basis. These three population-based datasets are routinely used in Canada to conduct health services research. Validation studies have shown them to have excellent sensitivity and specificity for identifying colorectal-related procedures and have high face validity for diagnosis codes [14–16]. Health services related to CRC diagnosis were defined using both procedure and diagnosis codes. Visits with procedure codes of: 1) diagnostic imaging to the abdominal or GI region including computerized tomography, ultrasound, X-ray or barium enema, or 2) lower endoscopy including colonoscopy and sigmoidoscopy were defined as related to CRC diagnosis. Physician visits with an ICD-9 or ICD-10 diagnosis code for any of: rectal bleeding, anaemia, abdominal pain, obstruction, abscess/fistula, constipation, perforation, diarrhea, weight loss, or change in bowel habit were defined as CRC-related [17–19]. Visits with any of the above diagnostic codes were defined as visits with CRC-relevant symptoms. ‘Symptom visits’ that occurred in the absence of a CRC-related procedure were further classified by type of physician as follows: 1) ‘GI specialist’ included gastroenterology, internal medicine and general surgery; 2) ‘non-GI specialist’ included all other specialists; 3) ‘GP’ included general practitioners, primary care and family physicians; and 4) ‘unknown provider-type’ when provider code was missing.

Diagnosis codes were also used to calculate Charlson co-morbidity scores using ICD-9/10 codes in the year prior to colorectal cancer diagnosis as described by Deyo et al. and updated for ICD-10 codes developed by Quan et al. [20, 21]. The Charlson comorbidity index was originally developed to estimate death within one year of hospitalization. The Charlson-Deyo index considers 17 comorbid conditions each with an associated weight, ranging from 1 to 6, based on the adjusted risk of mortality. The sum of all the weights results in a single comorbidity score for a patient. The Charlson score for each patient was categorized into three groups: 0, 1 and ≥2 to indicate no, mild and moderate-to-severe comorbidities. Chronic gastrointestinal (GI) tract conditions were also identified using ICD-9/10 codes in the year prior to diagnosis including: irritable bowel syndrome, irritable colon, Crohn's disease, ulcerative colitis, enteritis and diverticular disease of intestine, ulcer of anus and rectum and chronic vascular insufficiency intestine. These conditions are distinct from the Charlson co-morbidities as they were selected based on discussion with the authors and additional primary care physicians regarding conditions that may complicate the diagnosis of CRC, as opposed to predicting mortality. Each patient was identified with presence or absence of GI-related comorbidities. The Usual Provider Continuity (UPC) index, a measure of continuity of care with a primary care physician, was derived based on GP visits in the two years prior to diagnosis [22]. Statistics Canada data from 2006 were used as a source of patients’ median household income for each census dissemination area (DA), an area including approximately 600 households.

### Definition of diagnostic interval

**Fig. 1:**
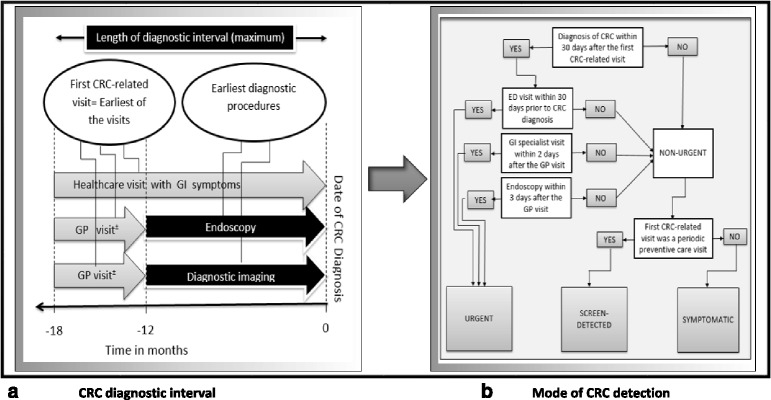
Illustrative flowchart for **a**) definition of CRC diagnostic interval and **b**) mode of CRC detection. CRC = Colorectal cancer, GI = Gastrointestinal, GP = General practitioner, ED = Emergency Department; ^±^GP visit refers to the most proximal GP visit prior to the first diagnostic imaging test or endoscopy exam

The diagnostic interval was defined as the time from the first CRC-related healthcare visit to the date of CRC diagnosis, as suggested by the Refined Anderson Model of Total Patient Delay within the constraints of the administrative data used for the study [23]. The first CRC-related healthcare visit was defined as the earliest of three record dates: 1) the most proximal GP visit prior to the first diagnostic imaging test, 2) the most proximal GP visit prior to the first endoscopy, or 3) the first date of GI symptoms (Fig. 1a). For (1) and (2), the date of the most proximal GP visit prior to the respective procedure was assumed to be the date the patient received a referral for the procedure. This assumption was made because in Alberta patients very seldom receive a specialist procedure or see a specialist without a referral. The referral usually comes from a GP. The exception to this is if a patient presents in an urgent state to an emergency department (ED). For this reason, if the first diagnostic imaging or endoscopy was performed in an ED and the patient did not have any prior record of a healthcare visit with GI symptoms then the ED visit was assumed to be the first healthcare contact. A one-year look-back period from the date of CRC diagnosis was used to identify relevant diagnostic imaging tests and endoscopies based on a sensitivity analysis we conducted that found roughly the same number of tests 12, 18, or 24 months prior to colorectal cancer diagnosis. A 6-month look-back period from date of diagnostic imaging or endoscopy was used to identify the GP referral visit – based on the recommendation by the Canadian Association of Gastroenterology [24] for the target maximal wait time from referral to procedure completion in absence of a symptom. To be consistent with the maximum timeframe for GP visits prior to diagnosis, an 18-month look-back period was used to identify all records with GI-related symptoms.

### Definition of mode of CRC detection

The study patients were classified by their mode of CRC detection: urgent, screen-detected or symptomatic, depicted in Fig. 1b. Presentation was defined as ‘urgent’ if the diagnostic interval was less than 30 days and the patient had any of the following: 1) an ED visit within the diagnostic interval; 2) a GI specialist visit within 2 days after the GP visit; or 3) an endoscopy performed within 3 days after the GP visit. The rationale for (2) and (3) is based on recommendations from the Canadian Association of Gastroenterology which state that emergency cases should be seen and endoscopy performed within 24 h following referrals [25]. To allow timing to contact the specialist and preparation for endoscopy, we extended this to 2 and 3 days, respectively. Patients were classified as screen-detected if their first CRC-related healthcare visit was a periodic preventive care visit with a GP, identified by the corresponding procedure code in the Physician Claims data, and they had not been classified as “urgent”. At that time, Alberta did not have a formal colorectal cancer screening program, thus the only screening that occurred was opportunistic, typically initiated at periodic preventive care visits. Many Canadians have a preventive care visit, typically once every year, to their family physicians, which is paid by the government’s universal healthcare system. Unlike usual primary care, a periodic preventive care visit allows individuals to undertake screening tests recommended based on the patient age, sex, family history, etc. The remaining patients were categorized as “unscreened”, and are, by exclusion, non-urgent and likely symptomatic.

### Statistical analysis

The primary outcomes were mode of CRC detection and time to diagnosis. Descriptive statistics were calculated to describe the association of patient demographics, clinical characteristics and healthcare utilization by mode of CRC detection: urgent; screening; or symptomatic. Healthcare utilization factors were categorized based on the frequency distribution of each variable. Chi-square test for categorical variables and Jonckheere-Terpstra trend test for ordinal variables were used to assess statistical significance.

By definition, the length of the diagnostic interval for urgently diagnosed patients is ≤30 days, therefore, this patient group was excluded from the analyses examining factors related to time to diagnosis. The median and 75^th^ percentile time to diagnosis in days was calculated for each variable of interest. The Kruskal Wallis test was used to assess for differences across medians within each categorical variable. Multivariable quantile regression was used to ascertain the association of demographic, clinical and healthcare utilization factors to the diagnostic interval (days). Quantile regression was used rather than the more typically used ordinary least square regression (OLS) model because the former allows examination of the way factor-effects change with increased time to diagnosis. Unlike OLS, quantile regression makes no distributional assumption about the error term in the model, and it offers considerable model robustness. Furthermore, the interpretation of the estimates is the difference in days associated with each factor relative to the reference group, making it simple to understand. All factors evaluated in the unadjusted analysis were included in the quantile regression except the number of diagnostic imaging tests and unknown provider-type to address issues of co-linearity. Backward elimination method was used to identify the most parsimonious regression model for the 50^th^ and 75^th^ percentiles.

All statistical analyses were performed using statistical software SAS 9.3 (SAS Institute, Cary, NC, USA).

## Results

A total of 11,241 patients were diagnosed in Alberta with colorectal cancer from 2004 to 2010. There were 1,615 patients excluded from the study for the following reasons: 477 had stage 0 cancer or a cancer histology that was not a CRC solid tumor; 467 had unknown stage; 229 did not have pathologic or microscopic confirmation of disease; 184 had a prior history of CRC or stomach cancer; 134 were diagnosed with another cancer within 6 months prior to the diagnosis of CRC; and 124 had no record of healthcare visits within 1.5 year prior to CRC diagnosis preventing estimation of time to diagnosis. The study cohort included the remaining 9,626 patients.

### Patients by mode of CRC detection and related factors

The median age of patients at diagnosis was 69 years and 57% were male. Approximately 25% of patients (2428) presented as urgent, 32% (3083) were screen-detected and 43% (4115) were non-urgent symptomatic. Table 1 shows the distribution of patient demographic and clinical characteristics by mode of CRC detection. Compared to screen-detected and symptomatic patients, urgent patients were significantly more likely to be older than 80 years, have colon rather than rectal cancer, stage IV tumor and a Charlson comorbidity score of 2 or higher but not have GI-related comorbidities. The screen-detected patients were most likely to be between 60 and 79 years, not have comorbidities, and reside in one of the two major cities, Calgary or Edmonton.

**Table 1:** Patient and clinical characteristics by mode of colorectal cancer diagnosis (*n* = 9626)

	Urgent	Screen-detected	Symptomatic	*p*-value^a^
	(*n* = 2428)	(*n* = 3083)	(*n* = 4115)	
	*n* (%)	*n* (%)	*n* (%)	
Patient characteristics				
Age at diagnosis				
< 50	228 (9)	211 (7)	383 (9)	0.0429
50–59	461 (19)	519 (17)	757 (18)	
60–69	586 (19)	792 (26)	1015 (25)	
70–79	599 (25)	1011 (33)	1230 (30)	
≥ 80	554 (23)	550 (18)	730 (18)	
Residential zones^b^				
Calgary	804 (33)	1084 (35)	1226 (30)	<.0001
Central	334 (14)	357 (12)	741 (18)	
Edmonton	836 (34)	1088 (35)	1320 (32)	
North	274 (11)	292 (9)	463 (11)	
South	180 (7)	262 (9)	364 (9)	
Median annual household income				
< $45389	647 (27)	705 (23)	993 (24)	0.4062
$45389 - $59119	590 (24)	681 (22)	1074 (26)	
$59119 - $80148	595 (25)	789 (26)	964 (23)	
≥ $80148	547 (23)	837 (27)	962 (23)	
Missing	49 (2)	71 (2)	122 (3)	
Clinical characteristics				
Anatomic site				
Colon	1672 (69)	1943 (63)	2471 (60)	<.0001
Rectum	756 (31)	1140 (37)	1644 (40)	
Stage at diagnosis				
I	280 (12)	781 (25)	886 (22)	<.0001
II	670 (28)	831 (27)	1150 (28)	
III	701 (29)	879 (29)	1191 (29)	
IV	777 (32)	592 (19)	888 (22)	
Charlson comorbidity score				
0	1359 (56)	1985 (64)	2528 (61)	0.0025
1	601 (25)	675 (22)	893 (22)	
≥ 2	468 (19)	423 (14)	694 (17)	
Gastrointestinal comorbidities				
Yes	544 (22)	827 (27)	1062 (26)	0.0005
No	1884 (78)	2256 (73)	3053 (74)	

^a^Jonckheere-Terpstra trend test for ordinal variables (age, income, comorbidity score and stage at diagnosis) and Chi-square test for the other categorical variables were used to compare proportions. ^b^One patient with unknown residential zone was excluded from this calculation

Table 2 shows the relationship between mode of CRC detection and healthcare utilization. Screen-detected patients were most likely to have medium to high continuity of usual provider care and patients who presented urgently were least likely, 79% versus 63%, respectively. A small proportion of patients, 5–7%, had one or more visits with a symptom to a non-GI specialist, regardless of mode of CRC detection. Non-urgent symptomatic patients had the highest number of total physician visits prior to diagnosis, with 41% having three or more visits compared to 33% of screen-detected and 15% of urgent patients; the increase was primarily due to larger numbers of GP visits with 16, 11 and 4% of non-urgent symptomatic, screened and urgent patients, respectively, having 3 or more GP visits.

**Table 2:** Healthcare utilization by mode of colorectal cancer diagnosis (*n* = 9626)

Healthcare Utilization	Urgent	Screen-detected	Symptomatic	*p*-value^a^
	(*n* = 2428)	(*n* = 3083)	(*n* = 4115)	
	*n* (%)	*n* (%)	*n* (%)	
UPC index				<.0001
<3 primary care visits	276 (11)	76 (2)	159 (4)	
Low (<50%)	620 (26)	586 (19)	904 (22)	
Medium (50%–80%)	821 (34)	1129 (37)	1440 (35)	
High (>80%)	711 (29)	1292 (42)	1612 (39)	
Physicians visits with GI-related symptom				
Visits to any physician				
0–1	1437 (59)	1063 (34)	1138 (28)	<.0001
2	631 (26)	1008 (33)	1302 (32)	
3	218 (9)	452 (15)	767 (19)	
4	81 (3)	240 (8)	365 (9)	
≥ 5	61 (3)	320 (10)	543 (13)	
GP visits				
0–1	2093 (86)	1859 (60)	2235 (54)	<.0001
2	254 (10)	856 (28)	1230 (30)	
3	44 (2)	200 (6)	333 (8)	
≥ 4	37 (2)	168 (5)	317 (8)	
GI specialist visits				
0	1761 (73)	2106 (68)	2771 (67)	<.0001
1	538 (22)	751 (24)	990 (24)	
≥ 2	129 (5)	226 (7)	354 (9)	
non-GI specialist visits				
0	2266 (93)	2918 (95)	3868 (94)	0.4904
≥ 1	162 (7)	165 (5)	247 (6)	
Unknown provider-type visits				
0	1822 (75)	2411 (78)	2975 (72)	<.0001
1	528 (22)	462 (15)	777 (19)	
≥ 2	78 (3)	210 (7)	363 (9)	
Visits for GI-related Procedure				
Endoscopies				
0	468 (19)	206 (7)	340 (8)	<.0001
1	1657 (68)	2360 (77)	3051 (74)	
≥ 2	303 (12)	517 (17)	724 (18)	
Diagnostic imaging tests				
0	1092 (45)	1551 (50)	1893 (46)	0.0001
1	1002 (41)	917 (30)	1270 (31)	
2	247 (10)	381 (12)	589 (14)	
≥ 3	87 (4)	233 (8)	362 (9)	

*Abbreviations*: *UPC* Usual provider care, *GP* General practitioner, *GI* Gastrointestinal, ^a^Jonckheere-Terpstra trend test was used to compare proportions

### Time to diagnosis

Table 3 shows the median and 75^th^ percentile time to diagnosis for the non-urgent patients by the demographic, clinical and healthcare utilization factors examined. The overall median and 75^th^ percentile time to diagnosis was 79 and 206 days, respectively. The median time for screen-detected patients was 74 days (interquartile range (IQR) 36–183 days) compared to 84 days (IQR 39–223 days) for symptomatic patients. The oldest patients, those > 80 years, had the longest diagnostic interval, with a median of 105 days, while patients aged 60–69 years had the shortest median at 69 days. The clinical factor that had the largest relationship to the length of the diagnostic interval was Charlson comorbidity index: those with a score of 2 or more had a median of 147 days compared to 70 days for those with a score of 0. In general, healthcare utilization factors affected the length of the diagnostic interval much more than patient demographic or clinical factors. The median diagnostic interval for patients with 5 or more CRC-symptom-related visits was significantly longer than the median for those with 0 or 1 CRC-symptom-related visits, 287 days and 38 days, respectively. Similarly, the median diagnostic interval for those with 3 or more CRC-symptom-related visits to a GP was 224 days compared to 56 for those with 0 or 1 visits and 224 days for those with 2 or more CRC-symptom-related visits to a GI specialist compared to a median of 102 days for those with 1 visit to a GI-specialist.

**Table 3:** Median and 75^th^ percentile of time to diagnosis of non-urgent colorectal cancer patients (*n* = 7198)

Factors	Time to diagnosis (days)		
	Median	75^th^ percentile	*p*-value^a^
Overall	79	206	
Patient and clinical factors			
Age at diagnosis			
< 50	81	177	<.0001
50–59	74	158	
60–69	69	172	
70–79	82	223	
≥ 80	105	286	
Residential zones			
Calgary	78	196	0.275
Central	84	227	
Edmonton	80	208	
North	78	223	
South	74	194	
Median annual household income			
< $45389	80.5	234	0.0035
$45389 - $59119	87	219	
$59119 - $80148	77	196	
≥ $80148	74	177	
Missing	74	278	
Anatomic site			
Colon	87	227	<.0001
Rectum	70	171.5	
Stage at diagnosis			
I	90	206	0.0007
II	78	212	
III	78	211	
IV	70	196.5	
Charlson comorbidity score			
0	70	164	<.0001
1	88	219	
≥ 2	147	322	
Gastrointestinal comorbidities			
Yes	88	195	0.0039
No	76	210	
Healthcare utilization factors			
UPC index			
< 3 primary care visits	50	112	<.0001
Low (<50%)	90	231	
Medium (50%–80%)	87	223	
High (>80%)	73	181.5	
Visits to any physician			
0–1	38	76	<.0001
2	71	151	
3	125	276	
4	189	348	
≥ 5	287	436	
GP visits			
0–1	56	127	<.0001
2	104	245	
≥ 3	226	384	
GI specialist visits			
0	64	160	<.0001
1	102	245	
≥ 2	224.5	413	
non-GI specialist visits			
0	76	194	<.0001
≥ 1	201	358	
Endoscopies			
0	97	271	<.0001
1	71	176	
≥ 2	129	285	
Diagnostic imaging tests			
0	61	135	<.0001
1	84	226	
2	125	290	
≥ 3	183	302	
Method of detection			
Screen-detected	74	183	0.0002
Symptomatic	84	223	

^a^Kruskal-Wallis test was used to compare medians across the groups

Table 4 presents the results of the quantile regression models at the 50th and 75^th^ percentiles.

**Table 4:** Quantile regression estimates of the median and 75^th^ percentile of the diagnostic interval for non-urgent colorectal cancer patients (*n* = 7198). The estimate associated with each category is the difference in days when compared to the reference category

	Q0.50		Q0.75	
	Days	*P*-value	Days	*P*-value
Intercept	7.28	0.89	15	0.08
Age at diagnosis				
< 50	2.33	0.5	−0.96	0.92
50–59	4.85	0.09	3.39	0.58
60–69	0	.	0	.
70–79	5	0.0425	8.59	0.17
≥ 80	14.27	0.0001	34.28	0.0003
Residential Zones				
Calgary	0	.	0	.
Central	3.18	0.33	9.23	0.25
Edmonton	−0.62	0.81	1.13	0.85
North	0.98	0.79	8.67	0.44
South	−7.15	0.0189	−15.33	0.0455
Anatomic site				
Colon	6.47	0.0014	9.56	0.0536
Rectum	0	.		
Stage at diagnosis				
I	25.67	<.0001	42.13	<.0001
II	8.47	0.0013	14.03	0.031
III	11	<.0001	23.98	0.0001
IV	0	.	0	.
Charlson Comorbidity Score				
0	0	.	0	.
1	5.13	0.0371	19.21	0.0025
≥ 2	31.86	<.0001	82.24	<.0001
GI comorbidity				
No	6.4	0.0014	29.18	<.0001
Yes	0	.	0	.
Usual Provider Continuity				
< 3 primary care visits	4.52	0.35	13.54	0.39
Low < =50%	5.47	0.07	18.34	0.0046
Medium 5080%	4.87	0.06	11.64	0.0269
High >80%	0	.	0	.
# of GP visits				
0–1	0	.	0	.
2	44.13	<.0001	91.45	<.0001
≥ 3	140.8	<.0001	201.59	<.0001
# of GI specialist visits				
0	0	.	0	.
1	33.46	<.0001	50.68	<.0001
≥ 2	108.54	<.0001	153.56	<.0001
# of non-GI specialist visits				
0	0	.	0	.
≥ 1	81.55	<.0001	101.56	<.0001
# of endoscopy				
0	17.44	<.0001	52.66	<.0001
1	0	.	0	.
≥ 2	36.05	<.0001	50.43	<.0001

The unconditional percentiles corresponded to diagnostic interval cut-off values are Q0.50 = 79 and Q0.75 = 206 days

The results are qualitatively similar to the unadjusted analysis shown in Table 3. The demographic and clinical factors with the largest effect were age: median diagnostic interval for oldest patients was 14 days longer than those aged 60–69 years; stage: median diagnostic interval for stage 1 patients was 26 days longer than stage IV patients; Charlson comorbidity score: median diagnostic interval was 32 days longer for those with a score of 2 or more compared to those with a score of 0.

The number of GP and GI specialist visits for CRC symptoms had the largest impact on the length of the diagnostic interval after adjusting for demographic, clinical and other healthcare utilization factors. Compared to patients with 0–1 GP visit with CRC symptoms, the median diagnostic interval for those with 2 or ≥3 was 44 and 141 days longer, respectively. Similarly, compared to patients who did not have any visits for CRC symptoms to a GI specialist, those who had 1 or ≥2 visits had a median diagnostic interval that was 33 and 108 days longer, respectively. Having any CRC-symptomatic visits to a non-GI specialist, however, was associated with almost 3 months longer median time to diagnosis.

## Discussion

In Alberta, Canada, a province with universal health coverage but without a formal colorectal screening program at the time of the study, 25% of patients presented as urgent while 20% had a diagnostic interval over 6 months, representing 45% of all newly diagnosed CRC patients over a 6-year period. Tørring et al. has shown that CRC patients with very short or very long diagnostic intervals have higher mortality [10]; efforts are clearly needed to improve the CRC diagnostic care process. Patients most likely to present as urgent were 80 years or older, have colon cancer, stage IV disease, and have multi-morbidities. The demographic and clinical factors that had the greatest impact on the length of the diagnostic interval were age greater than 80 years, stage I disease, and presence of multi-morbidities. Healthcare utilization factors had a much larger effect on the length of the diagnostic interval than clinical factors, however, with each GP or GI specialist visits for symptoms adding more than a month to the median time and a visit to a non-GI specialist for symptoms adding almost 3 months to the median time. Colon cancer is often difficult to diagnose, with non-specific symptoms developing slowly, whose import may be difficult for the patient to recognize, or require investigation or initial referral to a non-GI specialist. Therefore it is possible that many of these patients presented with non-specific findings that led to indirect routes of investigation and referral, before the correct diagnosis was determined. Regardless, these findings suggest that inefficiencies in the healthcare system are the most critical remediable factors to address.

A total of 32% patients identified as screen-detected via periodic preventive care check-up. Results from a 2009 survey showed that 43% of Albertan’s aged 50–74 years were up-to-date with their CRC screening [26]. This is reasonable given that our study included patients with CRC of all ages, whereas the screening rate is low in younger population. One possible improvement is to introduce a formal CRC screening program to decrease the percentage of patients presenting as urgent [27]. Regular screening addresses the difficulty of non-specific presentations by identifying cancer before symptoms begin. Countries with a national screening program, such as Germany, Italy and Australia, have reported urgent presentation rates ranging from 6 to 19% [28–30] significantly less than 25% found in the current study. Building awareness among patients and practitioners in understanding and recognizing the potential seriousness of symptoms is equally important. Educational efforts in combination with routine screening would help to mitigate patient- and practitioner-related factors of late presentation as well as clinical complexities due to non-specific symptoms [31, 32].

The patient population at highest risk for urgent presentation was those greater than 80 years with multi-morbidities. Likewise, these patients were also at highest risk for a long diagnostic interval. Other studies have also found that older patients and those with comorbidities have long diagnostic intervals; this may be due to missed opportunities related to non-specific symptoms, signs, and abnormal tests [33]. Providers may also be distracted from investigating GI symptoms or be hesitant to refer older patients to colonoscopy because of the higher complication rate in this population. Even with a screening program, cases will still present among the old, as will those not detected by screening. Therefore, the health care system must still identify and manage such patients presenting with symptoms.

The median length of the diagnostic interval was 79 days and the 75^th^ percentile was 206 days for screen-detected and symptomatic patients in our study. Two other population-based studies conducted in Canada that included slightly older patient cohorts, diagnosed in years 2001–2005, reported a median diagnostic interval of 44–64 days (depending on year of diagnosis) [12] and 44 days [34]. The two studies used similar methods to ours, however, an important difference is that we excluded urgent patients from the calculation of the diagnostic interval whereas the other two did not. If we include urgent patients, the median diagnostic interval was 55 days in our study with 37% of all patients diagnosed within 4 weeks, similar to 37.1% found by Porter et al. [34]. A multicenter US study that included mainly male patients of Veteran Affairs hospitals reported a median diagnostic interval of 91 days for screen-detected and 74 days for symptom-detected CRC [33]. Another observational study in Northern Holland reported a median diagnostic interval of 23.5 weeks (i.e., 165 days) using prospectively collected data [2]. The median diagnostic interval that the present study reported is similar to Fisher et al. [33], but much shorter compared to the other non-Canadian studies [2]. This difference may be partly due to differences in patient populations and study design but could also reflect differences in the healthcare systems.

Among the healthcare utilization factors, the increased number of GI-related healthcare visits for pre-diagnostic work-up had the greatest impact on the length of the diagnostic interval. If the diagnostic interval comprises discrete intervals, the primary care interval and the secondary care interval, as proposed by Olesen et al. [35], this finding is particularly interesting given our methods likely underestimate the length of the primary care interval since our start date is likely the date of referral from primary to secondary care. The fact that there were a higher proportion of patients with multiple GP visits than specialist visits suggests that the primary and secondary care intervals overlap in our study population significantly more than the ideal diagnostic care trajectory for a significant proportion of the population. While multiple visits may adversely affect time to diagnosis and the experience of care, our data do not enable us to identify the reasons for multiple visits to physicians: clinical complexity of the disease, misleading symptoms, reasonable watchful waiting, delay in appropriate investigations to confirm diagnosis, or possibly suboptimal professional performance of healthcare providers. A recent review suggests that diagnostic difficulty and the need for investigation of poorly differentiated symptoms in primary care are more likely to be the drivers for multiple visits than poor diagnostic reasoning and suboptimal professional practice [36]. Patient factors, including cancer awareness, beliefs and attitudes before presentation to healthcare system, are also factors contributing to delayed diagnosis [37]. On the contrary, others have found that poor physical examination and misdiagnosis by both primary and hospital physicians might contribute to repeated visits and lengthen time to diagnosis [38]. Increased healthcare utilization may also be due to 1) GP’s perception of needing more follow-up visits prior to referring for endoscopy, 2) management during the wait for “non-urgent” specialist consultation and 3) lack of service integration that would allow provision of multiple services at the same patient visit by the same healthcare provider or clinical team [12, 39].

### Strengths and limitations

Several limitations of the current study should be acknowledged. First, there will be a proportion of misclassification of the CRC presentation groups: urgent, screened, and symptomatic. We purposefully used a conservative definition for classifying urgent patients to minimize the chance of overestimation, however, in the absence of procedure codes indicating purpose of a test (i.e., screening or diagnostic) there will be misclassification in the screened and symptomatic groups, specifically, it is likely symptomatic patients who were identified at a preventive care visit are included in the ‘screened’ group. Second, it is not possible to determine whether patients presented urgently because they ignored early symptoms or because of errors and/or inefficiencies in the healthcare system, Similarly, it is not possible from this study to ascertain the extent of delays between physician appointments and tests due to patient lack of urgency, those due to system inefficiencies, or those due to valid clinical difficulty in diagnosis. In identifying time to diagnosis, we restricted the length of time to a maximum 18 months. This may result in underestimating the length of time to diagnosis for certain individuals given that a few patients may have presented to GP prior to the first contact we identified but is unlikely to have affected the median or 75^th^ percentile, both of which were considerably less than 18 months.

A major strength of the study is that it is population-based and included all histologically confirmed incident colorectal cancer cases over a 7-year period, minimizing the potential for selection bias. Quantile regression allowed us to estimate the effect factors of interest had directly on the length of the diagnostic interval, with respect to the median and 75^th^ percentile. We also used an innovative definition of urgent presentation by combining multiple events of urgent nature (i.e., time to diagnosis, time and status of ED visit, endoscopy exams and specialist visit).

### Implications and areas for further research

The relationship between timeliness of care and factors associated with the pre-diagnostic healthcare delivery is complex. Actions are needed to reduce the frequency of urgent presentation as well as improve the timeliness of diagnosis. Both of these can be addressed to some extent by implementation of an organized screening program in the eligible general population, however, many cases will continue to present despite the program, and systems must assist these patients too. The effect on resource utilization is critical when developing and implementing an organized screening program, which may increase number of diagnoses [40, 41]. The goal of reducing time to diagnosis can also be achieved by better service integration and coordination of care across service providers [39].

## Conclusion

Major efforts are needed to 1) identify CRC at an early stage when it is treatable, for instance, through an organized screening program and 2) to develop, test, and implement care models that address the healthcare needs of those with multiple morbidities in a timely fashion. A province-wide colorectal screening program has been recently instituted; the current study provides strong baseline data that can be used to evaluate the screening program’s effectiveness in decreasing the frequency of urgent presentation and improving timely diagnosis.

## Abbreviations

CRC: Colorectal cancer
DA: Dissemination area
ED: Emergency department
GI: Gastrointestinal
GP: General practitioner
ICD-O: International classification of disease for oncology
IQR: Interquartile range
OLS: Ordinary least square regression
UPC: Usual provider continuity
